# Reliability and validity of a Central Kurdish version of the Dizziness Handicap Inventory

**DOI:** 10.1038/s41598-019-45033-1

**Published:** 2019-06-12

**Authors:** Sherko Saeed F. Zmnako, Yousif Ibrahim Chalabi

**Affiliations:** Department of Surgery-Otolaryngology, College of Medicine, University of Sulaimani, Sulaimani, 46001 Kurdistan Region, Iraq

**Keywords:** Diseases, Medical research

## Abstract

We cross-culturally adapted the Dizziness Handicap Inventory (DHI) into Central Kurdish dialect (DHI**−**CK) and verified its reliability and validity. A cross-sectional study was utilised to measure the impacts of vestibular disorders. Along with the DHI**−**CK, two comparators were introduced: the Visual Analogue Scale and the Clinical Test of Sensory Interaction and Balance. External and internal reliability were tested with intraclass correlation coefficient (ICC) and Cronbach’s alpha/composite reliability, respectively. Patients (n = 301; mean age = 44.5 ± 15.2 years; 59.8% women) presenting with vestibular symptoms for at least 30 days who were diagnosed with a vestibular disorder and healthy participants (n = 43; mean age = 42 ± 17.9 years; 62.8% women) (N = 344). The DHI**−**CK and its three sub-scales—Physical, Emotional, Functional—exhibited good to excellent external reliability: ICCs in the test-retest were 0.93, 0.88, 0.91, and 0.92, respectively. Cronbach’s alphas were 0.87, 0.71, 0.75, and 0.73, respectively. Convergent validity was supported by Spearman’s correlations between the DHI**−**CK and the comparators. The receiver operating characteristic curve analysis confirmed discriminating validity. The DHI**−**CK was cross-culturally validated. It is a reliable and valid tool that can be used by clinicians and researchers to quantify vestibular disorder outcomes in Kurdish**-**speaking populations.

## Introduction

Vestibular symptoms are common and are associated with major health and cost issues^1^. Patients with vestibular disorders require frequent visits to primary care centres^2^; furthermore, their assessment is challenging, and the symptoms and consequences produced by these disorders are imprecise, subjective, and difficult to study and quantify^3^. Objective findings such as caloric tests, laboratory results, and even radiological investigations are of limited value if they do not coincide with clinical findings^4^. Therefore, over the past few decades, researchers and clinicians presented a satisfactory solution to quantify the symptoms through development of suitable instruments: patient-reported outcome measures (PROMs), which are typically complete via self*-*administered questionnaires. PROMs are a quick, authentic way to measure the impacts of demanding disorders^5,6^.

However, for PROMs to be qualified, they must be reliable; otherwise, performing clinical research and/or practice with instruments of poor quality is unethical and a waste of resources^7^. The outcome data of any measurement-instrument are trustworthy only if that instrument has been academically subjected to reliability and validity testing^5^.

Translation of a valid instrument to another language may dissipate its quality because of cultural differences among populations. Therefore, in addition to translation and cultural adaptation, reliability and validity must also be repeated and reported in harmony with the noted guidelines^8^.

The Dizziness Handicap Inventory (DHI) was developed by Jacobson and Newman^9^. It is a widely used PROM in the vestibular field^10^. The 25-item tool comprises three sub-scales: physical (DHI‒P; 7 items), emotional (DHI‒E; 9 items), and functional (DHI‒F; 9 items). For each item, the respondent must select one of three responses, each assigned a specific value (*yes* = 4, *sometimes* = 2, and *no* = 0). The total sum of the scores in three sub-scales (DHI‒T) range 0–100, with higher score indicating greater self-reported handicap.

The original English version of the DHI has been cross-culturally validated in many other languages, including several languages that are spoken in the Middle East: Hebrew^11^, Arabic^12^, Persian^13^, and Turkish^14^. To our knowledge, there is no validated vestibular PROM in Kurdish; therefore, we cross-culturally adapted the DHI into Central Kurdish dialect (DHI‒CK) and verified its reliability and validity.

## Results

### The logic sequence of the study

**Figure 1:**
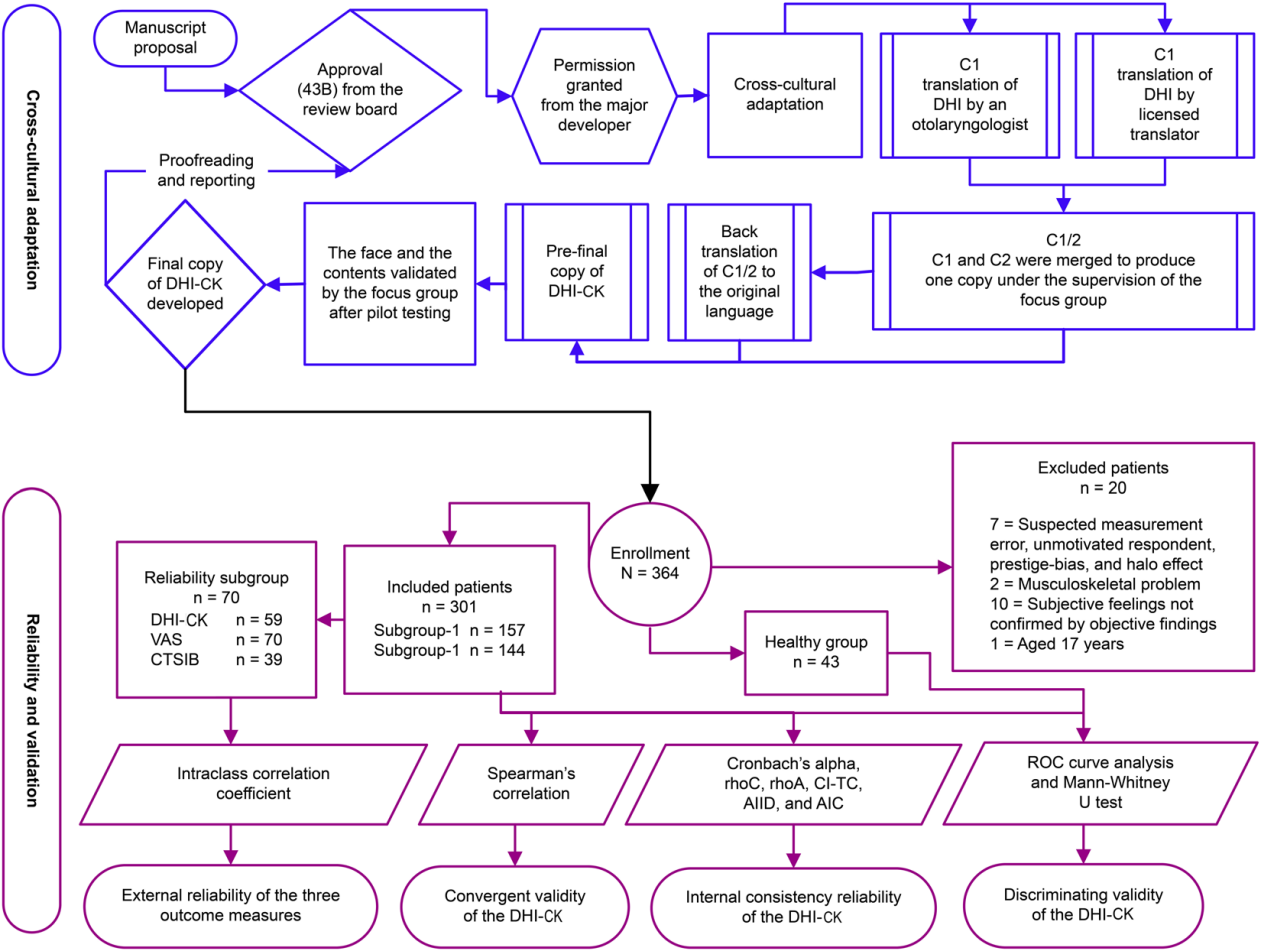
The logic sequence of the study. Abbreviations: C1, first translated copy; C2, second translated copy; C1/2, merge of C1 and C2; DHI‒CK, Dizziness Handicap Inventory‒Central Kurdish; VAS, Visual Analogue Scale; CTSIB, Clinical Test of Sensory Interaction and Balance; rhoC, composite reliability; rhoA, consistent reliability of the partial least squares; CI‒TC, corrected item-total correlation; AIC, average inter-item correlation; AIID, alpha if item deleted; ROC, receiver operating characteristic.

The flowchart in Fig. 1 demonstrates the steps of cross-cultural adaptation, enrolments, and the statistical approaches for assessment of psychometric properties of the DHI‒CK. Among the 321 patients, 20 were excluded; however, the exclusions did not result in significant differences in the analyses.

### Participants’ baseline characteristics

Table 1 provides participants’ baseline characteristics. Patients’ (n = 301; 59.8% women) mean age was 44.5 ± 15.2 years (range = 61 years). Healthy participants’ (n = 43; 62.8% women) mean age was 42 ± 17.9 years (range = 57 years). The percentage of patients in the three age ranges was as follows: n = 49, 16.3% (18–29 years); n = 187, 62.1% (30–59 years); and n = 65, 21.6% (60–79 years). Patients with no or only a primary education (n = 163; 54.2%) were assisted by an interviewer with survey completion. More than half of the patients (n = 157; 52.2%) had vestibular symptoms within the range of 1–6 months. The unilateral peripheral vestibular hypofunction was the commonest disorder (35.9%).

**Table 1:** Participants’ baseline characteristics (N = 344).

	Patients		Reliability subgroup		Duration subgroups^a^				Healthy group	
					Subgroup−1		Subgroup−2			
	n = 301		n = 70		n = 157		n = 144		n = 43	
	M	SD	M	SD	M	SD	M	SD	M	SD
Age (years)	44.5	15.2	45.8	16.5	43.4	15.5	45.7	14.8	42	17.9
Duration^a^	17.3	28.8	12.6	27.3	2.4	1.6	33.8	35		
	**n**	**%**	**n**	**%**	**n**	**%**	**n**	**%**	**n**	**%**
Women	180	59.8	34	48.6	89	56.7	91	63.2	27	62.8
**Education**										
No or Primary^bc^	163	54.2	42	60	86	54.8	77	53.5	22	51.2
Secondary^bd^	87	28.9	15	21.4	43	27.4	44	30.6	14	32.6
Higher education^de^	51	16.9	13	18.6	28	17.8	23	16	7	16.3
**Diagnosis**										
BPPV	41	13.6	7	10	23	14.6	18	12.5		
MD	24	8	10	14.3	8	5.1	16	11.1		
UPVH	108	35.9	27	38.6	64	40.8	44	30.6		
VM	26	8.6	3	4.3	15	9.6	11	7.6		
Other VD^f^	102	33.9	23	32.9	47	29.9	55	38.2		

Note: ^a^Subgroups categorised based on duration of vestibular symptoms in months: 1−6 and 7−180 months for subgroups 1 and 2, respectively; ^b^Schools; ^c^DHI-CK administered by an interviewer; ^d^DHI-CK administered by the patient; ^e^Education higher than secondary school, that is, diploma, bachelor, and postgraduate educations; ^f^Distinct diagnoses could not be recognised.

Abbreviations: M, Mean; SD, Standard Deviation; BPPV, Benign Paroxysmal Positional Vertigo; MD, Meniere’s Disease; UPVH, Unilateral Peripheral Vestibular Hypofunction; VM, Vestibular Migraine; VD, Vestibular Disorders; DHI-CK, Dizziness Handicap Inventory-Central Kurdish.

### External reliability

The four scales of the instrument revealed good to excellent external reliability; the intraclass correlation coefficients (ICC) of the test-retest reliability for DHI–P, DHI–E, DHI–F, and DHI–T were 0.88, 0.91, 0.92, and 0.93 respectively. The total scores of both comparators—clinical test of sensory interaction and balance (CTSIB–T) and Visual Analogue Scale (VAS–T)—also exhibited excellent reliability: 0.91 and 0.95, respectively (Table 2).

**Table 2:** External reliability of the three outcome measures.

	n = 59								n = 39		n = 70	
	DHI–P		DHI–E		DHI–F		DHI–T		CTSIB–T		VAS–T	
	ICC^a^	n	ICC^a^	n	ICC^a^	n	ICC^a^	n	ICC^a^	N	ICC^a^	n
Test-retest	0.88	59	0.91	59	0.92	59	0.93	59	0.91	39	0.95	70
Inter-interviewer	0.95	24	0.90	24	0.95	24	0.97	24	0.93	16	0.95	29
Intra-interviewer1	0.81	16	0.88	16	0.91	16	0.90	16	0.95	12	0.92	18
Intra-interviewer2	0.82	19	0.94	19	0.89	19	0.90	19	0.76	11	0.97	23

Note: ^a^Intraclass correlation: two-way mixed effects, mean of k interviewers, and absolute agreement for the model, type, and the definition, respectively.

Abbreviations: DHI–P/E/F/T, Dizziness Handicap Inventory–Physical/Emotional/Functional/Total, respectively; CTSIB–T, Clinical Test of Sensory Interaction and Balance–Total; VAS–T, Visual Analogue Scale–Total; ICC, Intraclass Correlation Coefficient.

### Internal consistency reliability

Cronbach’s alpha (**α**) s of the DHI–P, DHI–E, DHI–F, and DHI–T were 0.71, 0.75, 0.73, and 0.87, respectively. The average inter-item correlations (AIC) of all scales were satisfactory as they were located within the acceptable range of 0.2–0.5. The corrected item-total correlations (CI‒TC) of the 25 items in all scales showed acceptable values; nearly all the 25 items in the DHI–T acquired values above 0.3 (item-F7 was 0.29). Both composite reliability (rhoC) and reliability of the partial least squares (rhoA) in the three sub-scales were >0.7 (Table 3).

**Table 3:** Internal consistency variables of Kurdish, Original, and German versions.

	DHI–CK (Kurdish)				Original^a^	German^b^
	n = 301				n = 106	n = 127
	Corrected item-total correlation					
	DHI–P	DHI–E	DHI–F	DHI–T	DHI–T	DHI–T
P1- Looking up	0.48			0.31	0.54	0.32
E2- Being frustrated		0.41		0.42	0.34	0.51
F3- Restricting travel			0.54	0.51	0.76	0.61
P4- Walk via supermarket aisle	0.35			0.44	0.39	0.48
F5- Getting out or into bed			0.22	0.33	0.50	0.41
F6- Restricting social activities			0.52	0.53	0.69	0.72
F7- Reading difficulties			0.24	0.29	0.44	0.36
P8- Sports-like activities	0.38			0.52	0.54	0.67
E9- Afraid to leave home alone		0.47		0.50	0.43	0.49
E10- Embarrassment		0.40		0.46	0.46	0.27
P11- Quick head movement	0.58			0.47	0.51	0.41
F12- Avoid heights			0.22	0.32	0.49	0.42
P13- Turning over in bed	0.38			0.34	0.43	0.27
F14- Heavy housework			0.51	0.54	0.58	0.69
E15- Considered intoxicated		0.28		0.33	0.30	0.48
F16- Difficult to go for a walk			0.50	0.61	0.62	0.57
P17- Sidewalk walking	0.28			0.41	0.58	0.46
E18- Concentration difficulties		0.27		0.33	0.49	0.51
F19- Walking in the dark			0.28	0.35	0.48	0.32
E20- Fear of being alone		0.45		0.48	0.27	0.37
E21- Feeling handicapped		0.57		0.45	0.41	0.71
E22- Stress on relationships		0.50		0.49	0.46	0.60
E23- Being depressed		0.54		0.39	0.41	0.63
F24- Responsibility issues			0.58	0.63	0.56	0.66
P25- Bending over	0.50			0.46	0.57	0.32
Cronbach’s alpha	**0.71**	**0.75**	**0.73**	**0.87**		
AIC	0.26	0.25	0.22	0.22		
RhoC	0.80	0.82	0.80			
RhoA	0.71	0.76	0.77			

Note: For simplicity, items reduced; Alphas of the scales are in bold; ^a^Jacobson, G. P. & Newman, C. W. The development of the dizziness handicap inventory. *Arch. Otolaryngol. Head Neck Surg* **116**, 424−427, 10.1001/archotol.1990.01870040046011 (1990); Kurre, A. *et al*. Translation, cross-cultural adaptation and reliability of the German version of the dizziness handicap inventory. *Otol. Neurotol*. **30**, 359−367, 10.1097/MAO.0b013e3181977e09 (2009).

Abbreviations: DHI‒CK/P/E/F/T, Dizziness Handicap Inventory–Central Kurdish/Physical/Emotional/Functional/Total, respectively; AIC, Average Inter-item Correlation; rhoC, Composite reliability; rhoA, Consistent reliability of the partial least squares.

We estimated the **α** if item deleted (AIID); that is, the resulting **α**s of the sub-scales and the total scale when any item was deleted, no inflation was noticed in these **α**s.

In non-normal item-E15, the frequency of the 301 responses was as follows: yes = 16, sometimes = 11, and no = 274. The standardised values of each of the records were <3.29 except for those of yes-response records (3.88).The possible negative effects of this non-normality were investigated by analysing data with and without the item; however, almost all internal consistency parameters remained the same (Supplementary Table S1).

### Convergent validity

Spearman’s correlation between DHI–T and VAS–T was 0.64; correlations of CTSIB–T with DHI–P and DHI–F were −0.31 and −0.38, respectively (Table 4); similar results were provided by Pearson’s correlations (Supplementary Table S2).

**Table 4:** Spearman’s correlations between the scales and the comparators.

	n = 301			n = 290	n = 286
	DHI–P	DHI–E	DHI–F	VAS–T	CTSIB–T
DHI–P				0.46	**−0.31**
DHI–E	0.41			0.57	−0.30
DHI–F	0.67	0.69		0.58	**−0.38**
DHI–T	0.79	0.82	0.93	**0.64**	−0.39

Note: Correlations mentioned in the hypotheses are in bold.

Abbreviations: DHI–P/E/F/T, Dizziness Handicap Inventory–Physical/Emotional/Functional/Total, respectively; VAS–T, Visual Analogue Scale–Total; CTSIB–T, Clinical Test of Sensory Interaction and Balance–Total.

### Discriminating validity

**Figure 2:**
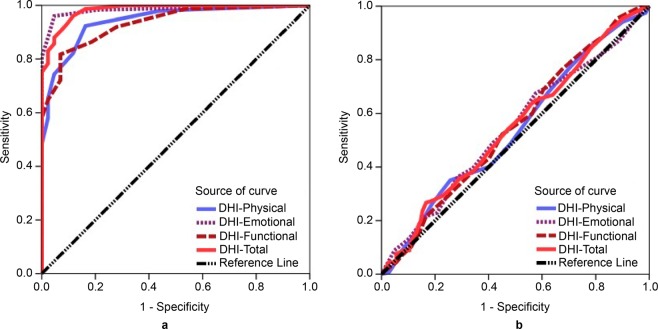
Comparison of receiver operating characteristic curves in different groups and subgroups. (**a**) Patient (n = 301)/heathy group (n = 43); (**b**) subgroup-1 (n = 157)/subgroup-2 (n = 144). Note: Subgroups categorised based on duration of vestibular symptoms in months: 1−6 and 7−180 months for subgroups 1 and 2, respectively. Abbreviation: DHI, Dizziness Handicap Inventory.

**Figure 3:**
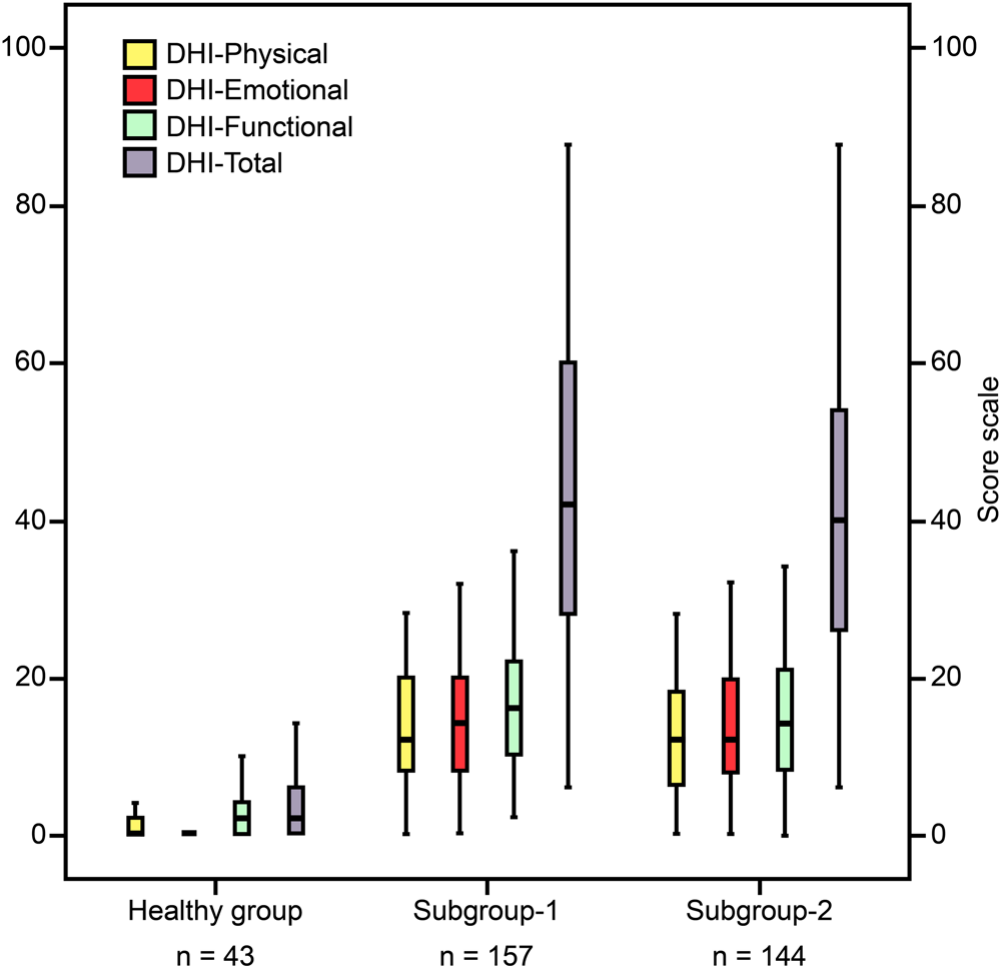
Shape and distribution of the scales in healthy and patients’ subgroups. Note: Subgroups categorised based on duration of vestibular symptoms in months: 1−6 and 7−180 months for subgroups 1 and 2, respectively. Abbreviation: DHI, Dizziness Handicap Inventory.

In patient/healthy groups, the areas under the receiver operating characteristic (ROC) curve (AUC) of the scores DHI–P, DHI–E, DHI–F, and DHI–T were 0.94, 0.98, 0.93, and 0.98 respectively; however, in patients’ subgroups were 0.54, 0.54, 0.55, and 0.55 respectively (Table 5 and Fig. 2). Moreover, the Mann-Whitney U test retained the null hypothesis when the scores of patients’ subgroups were compared with each other (*ps* > 0.05); however, it was rejected when the scores of all patients and their subgroups were compared with those of the healthy group (*ps* < 0.05) and distinct distributions and shapes in all sub-scales and the total scale were revealed (Fig. 3).

**Table 5:** The ability of the scales to discriminate between different groups and subgroups using receiver operating characteristic curve.

	Patient group (n = 301)					Patients’ subgroup-1^ab^ (n = 157)				
	Healthy group (n = 43)					Patients’ subgroup-2^ab^ (n = 144)				
	AUC	Youden index	Criterion value	Sensitivity	Specificity	AUC	Youden index	Criterion value	Sensitivity	Specificity
DHI-P	0.94	0.76	>2	92.36	83.72	0.54	0.09	>16	35.03	74.31
DHI-E	0.98	0.91	>2	96.01	95.35	0.54	0.10	>10	67.52	42.36
DHI-F	0.93	0.75	>6	81.73	93.02	0.55	0.09	>8	76.43	32.64
DHI-T	0.98	0.84	>10	96.01	88.37	0.55	0.10	>58	26.75	83.33

Note: ^a^Subgroups categorised based on duration of vestibular symptoms in months: 1−6 and 7−180 months for subgroups 1 and 2, respectively; ^b^Subgroup-1 and subgroup-2 defined as case and control, respectively.

Abbreviations: AUC, Area Under the receiver operating characteristic Curve; DHI-P/E/F/T, Dizziness Handicap Inventory-Physical/Emotional/Functional/Total, respectively.

## Discussion

Validated PROMs are of utmost importance when examining vestibular disorder; unfortunately, to date, there has been no such instrument in Kurdish that can quantify the impact of vestibular disorders. Accordingly, using a focus group and key recommendations, we cross-culturally adapted the DHI into Central Kurdish.

Convincing a patient to participate in the target population was not difficult—meticulous explanation of the potential benefits of this study by the authors and the interviewers (raters) likely increased the participation rate. However, maintaining participants’ motivation was challenging. We occasionally noticed that, after a few responses, participants’ interest declined, which was resolved by changing from self-administered to interviewer-administered. Hence, employing interviewers was essential. Interviewers were instructed to delineate bias scores in cases of unreliable respondents, prestige-bias (where the patient reports what s/he wants instead of what s/he feels), and halo-effects (where the patient overgeneralises the responses in either a positive or negative direction)^15^.

Dizziness is a broad term, and it might be of non-vestibular origin^16^; however, the DHI was originally developed to evaluate the consequences of vestibular disorders. Therefore, to ensure sample representativeness, we only included cases with a vestibular origin. Additionally, patients were of various ages from diverse settings.

The DHI‒CK and its three sub-scales showed good to excellent external reliability. The present study almost replicated the test-retest reliability of the original scale^9^, and other translated versions^13,17–19^. Further, the internal consistency was broadly examined through most of the recommended criteria, and the DHI‒CK and its three sub-scales had acceptable to good reliability. The CI‒TC values for each item in the DHI‒CK were compared with that of the original and German version^19^, which also revealed internal consistency (see Table 3). However, our cut-off point of 0.2 for the CI–TC (the same used for the German version) varied from those reported (e.g. 0.3, 0.4, and 0.5) by other guidelines^20,21^. If we consider this discrepancy and recall that the DHI was originally developed based on the CI‒TC, one could argue about the structure of this popular PROM. In other words, factor analysis is superior to CI‒TC when examining the structural organisation of sub-scales. This was tested by both Kurre and colleagues^22^ and Tamber and colleagues^18^; when they subjected DHI to a structural analysis, structures that differed from those of the original were found.

The non-normal E15 item (i.e. ‘are you afraid people may think you are intoxicated?’) and its effects on the analysis were thoroughly investigated. Concerning bias, a score related to alcohol consumption in a semi-conservative population (Kurdish) is a matter of debate. The possibility of prestige-bias in *no*-response records was considered, because this response is socially acceptable; likely the *yes*-response (potential outliers) provided legitimate data. Accordingly, it would be illogical to remove genuine data; further, deletion of these outliers makes the sample less representative. Consequently, to examine the effect of these aberrant 16 cases, instead of deletion, we analysed the data with and without item-E15. We planned to permanently remove the item from the DHI‒CK if there was substantial variation between the two analyses; however, no significant differences were found; therefore, the item was retained.

Our hypotheses regarding convergent validity were supported; an adequate positive correlation was found between the DHI‒T and VAS‒T, and a similar association was seen in the German version^19^. Furthermore, the negative and moderate range of correlations between the related sub-scales (DHI‒P and DHI‒F) and the objective score (CTSIB‒T) in this study were similarly generated by both Kurre and colleagues^19^ and Nikitas and colleagues^23^, by correlating distinct types of objective scores with the DHI sub-scales.

This study revealed that the duration of the symptoms did not significantly affect the DHI scores; the instrument could not discriminate subgroups with different elapsed time for symptoms, confirming that the scores are collective measures. However, the ROC curve analysis and default Mann-Whitney U test confirmed that the instrument can effectively discriminate between healthy individuals and patients with vestibular disorders.

This study had some limitations. First, there were no validated PROMs for vestibular specialty in Kurdish to be used as a comparator in this study. Second, the C/12 was back-translated only once. Lastly, the least time interval in reliability tests was reduced to one day because of patients’ housing situation. We noticed that long intervals are not suitable for reproducibility in patients with vestibular disorders because symptoms can change dramatically under the effect of central compensation; therefore, to avoid recall bias, it is better to use other measures, such as those that we mentioned in our Methods section.

Despite these limitations, we believe that this work provides an essential tool that can be used by clinicians and researchers when examining Kurdish-speaking populations with such demanding disorders; moreover, this tool can be used as a cornerstone and a comparator when validating other similar PROMs in the future.

The Kurdish medical community was deprived from any validated PROM in the field of vestibular disorders. Consequently, we cross-culturally adapted the DHI‒CK and verified its external and internal reliability. We also established that it had acceptable convergent and discriminating validity. As an effective PROM, the DHI‒CK can be utilised by clinicians and researchers to quantify the impacts of vestibular disorders in pre and post-therapeutic interventions. Further research should assess its internal dimensions, responsiveness, and interpretability.

## Methods

### Ethics

The present study commenced after obtaining approval (no. 43B) from the Ethical Review Board of the College of Medicine, Sulaimani University, Sulaimani, Kurdistan Region, Iraq. This study was conducted in accordance with the 2008 Declaration of Helsinki. Participants who met the inclusion criteria were enrolled after providing informed, written consent.

### Cross-cultural adaptation

Steps in this process were implemented per regulations provided by two related guidelines^8,24^.

### Initial stage

The initial stage comprised three steps:

1. Endorsement for cross-cultural adaptation to Kurdish was granted from professor Jacobson, the original developer^9^.
2. We ensured that translated questions were understandable. Words or expressions that are not familiar must be substituted by the most appropriate ones without losing their meaning.
3. We implemented necessary focus-group sessions (consisting of 7 otolaryngologists) according to specific guidelines^25,26^.

### Translation stage

The DHI was translated from English to Central Kurdish twice: the first copy (C1) by an expert otolaryngologist and the second (C2) by a professional bilingual translator. Both were synthesised to form C1/2. During synthesis, vague words were clarified, and formal expressions were popularised (e.g. ‘dancing’ was changed to ‘*shayi*’, which represents a traditional celebration; and the translated word for ‘embarrassed’ was replaced by a more popular Arabic word).

Then, the C1/2 was back-translated to English and compared with the original version—which revealed they were congruous—followed by minor editing for the pre-final copy. Next, a pilot study was conducted with 12 educated patients with good linguistic skills from the target population to clarify the questions, and the focus group was used to determine face and content validity. Eventually, after proofreading, the final copy was created (Supplementary Table S3), and the procedure was reported to the institute.

### Design and participants

A cross-sectional survey was utilised to perform the study; however, for the reliability subgroup, the survey was converted to a short-term longitudinal.

Enrolment occurred in two well-resourced tertiary clinics that cover a considerable amount of the Sulaimani governorate in Iraq. Participants’ cognitive state was assessed through a general clinical examination; additionally, for older participants (aged >65 years), the Mini-Mental State Examination was also utilised. Inclusion criteria were as follows: aged 18 to 79 years, having vestibular symptoms for at least 30 days, received an objective diagnosis of a vestibular disorder, and passing the cognitive assessment. Participants who could not answer or were unable to perform objective tests and those with associated non-vestibular pathology were excluded from analyses. Because of the duration of vestibular symptoms, included patients were categorised into two subgroups: 1 (symptoms for 1–6 months) and 2 (symptoms for 7−180 months).

The sample size was determined based on the participant-to-variable ratio of at least 10 participants for each item^27^. Accordingly, we estimated that 301 patients would be sufficient. From March 2017 to June 2018, patients were included in the study.

The DHI is a self-administered tool; therefore, the interviewer’s role was minimal^28^; however, because of the inclusion of illiterate participants, the survey involved two interviewers with proximate abilities. The job of the interviewers was to introduce the task, provide any necessary explanations, and/or read the items to participants who could not read.

Patients in the reliability subgroup (n = 70), were rated on two occasions. The interval between occasions was 1 to 5 days for both PROMs; while, for the objective test—CTSIB—the interval was 1 to 2 hours (to avoid the effects of in-between rehabilitations and/or central adaptation). The time of the second rating was adjusted by the interviewers per patients’ availability.

### Randomisation process

While patients were receiving the results of their tests or rehabilitation treatments, they were invited to participate. Those who consented and met the inclusion criteria were systematically numbered. The first patient was selected randomly followed by a constant interval selection.

### Measurement errors and recall bias

Steps were taken to minimise measurement errors and recall bias such as changing the sequence of the questions, applying a similar setting, excluding unstable patients, and not interfering with the patients during response selection.

### Comparator instruments

In addition to the DHI‒CK, two other outcome measures were introduced.

### VAS

The VAS has been widely used as an outcome measure. de Boer and colleagues^29^ concluded that the VAS has good psychometric properties. Because of the lack of any validated PROMs in Kurdish that can measure the same construct, we utilised the VAS as a comparator. A printed scale with one-hundred fractions from zero to 100 was used: in which, zero denotes no-handicap and 100 denotes maximum-handicap. Patients were asked to score his/her overall resultant handicap (VAS‒T) since vestibular symptom onset.

### CTSIB

Participants were asked to maintain balance for three trials in six conditions. They were standing with both legs and feet close together, wearing socks, and looking forward with each palm over the corresponding shoulder. The six conditions were as follows: (1) stable and flat surface with eyes open, (2) stable and flat surface with eyes-closed, (3) stable and flat surface with eyes-open and wearing a visual-conflict dome, (4) compliant spongy surface with eyes open, (5) compliant spongy surface with eyes closed, and (6) compliant spongy surface with eyes open and wearing a visual-conflict dome. Any trial was completed if the participant could or could not maintain his/her balance for 1 minute, moving palm or foot, loss of balance, seeking assistance, or opening eyes in the eyes-closed condition. Second and/or third trials were only needed if the participant could not complete the 1 minute in the preceding trial. For each condition, the sum was calculated by dividing the total seconds for available trial/s on number of trial/s for that condition, while the CTSIB‒T was the total of all six conditions^30^.

### Hypotheses

DHI‒CK and the designed VAS for this study are subjective scores; they are cumulative measures for the same construct; i.e. the global handicap induced by vestibular disorders from the onset of symptoms to the time of rating. However, CTSIB‒T is an objective score that measures the steadiness at a specific time; i.e. the time of testing^31^. Appropriately, to assess the concept and the discriminating ability of the instrument on the base of the duration (elapsed time from the beginning of the symptoms to the time of rating), we categorised our patients into two subgroups and devised the following three hypotheses:

1. In all patients, the positive correlation between the DHI‒T and VAS‒T would be adequate;
2. In all patients, the negative correlation between CTSIB‒T with both DHI‒P and DHI‒F would be moderate because they are measuring the steadiness in two distinct ways (objective and subjective); and
3. The distribution of the four DHI scores (three sub-scales and total) would be the same across patients’ subgroups because the scores are a cumulative measure and are not related to the amount of time elapsed; however, it would differ between the all patients/subgroups and the healthy group because the tool was originally designed to measure the impacts of vestibular disorders.

### Statistical analyses

#### Data screening

Records with missing values were pair-wise excluded. Ceiling and floor effects were absent in the three outcome measures. Considering our sample size, an absolute value for standardised Z-score greater than 3.29^32^ and absolute values greater than 2 and 7 for skewness and kurtosis^33^ respectively, were considered as non-normal; moreover, a chi-square critical value of <0.001 in Mahalanobis distance was considered a multivariate outlier^34^.

The scores of 24 questions and the four scales were distributed normally, as none of them exceeded these cut-off points. However, the normality was violated by Item-E15, in which, absolute skewness and kurtosis were 3.32 and 9.7, respectively (Supplementary Table S1), and Z-scores of each of the 16 cases were 3.88 (>3.29); therefore, they were considered as a potential univariate outlier. Necessarily, we tested the multivariate distribution for all 25-items using IBM SPSS macro from DeCarlo^35^, which revealed asymmetry and significant p-values for both skewness and kurtosis (Mardia’s test). Non-normality is expected in ordinal data such as Likert-items^36^; consequently, we followed Feng *et al*.^37^ and utilised non-parametric tests instead of log-transformation.

#### External reliability

Because of the involvement of two specific interviewers, the choice of the model, type, and the definition of ICC were two-way mixed-effect, mean of k interviewers, and absolute agreement, respectively. Referenced values of <0.5, from 0.5 to 0.75, from 0.75 to 0.90, and >0.90 indicate poor, moderate, good, and excellent reliability, respectively^28^.

#### Internal consistency

The following six variables and their corresponding referenced values were used:

1. **α**, >0.7^38^.
2. AIC, from 0.2 to 0.5^39^.
3. CI‒TC, >0.2^21^.
4. AIID, when any item deleted, **α** of the corresponding scale should not inflate^38^.
5. rhoC, >0.7.
6. rhoA, >0.7^40^.

#### Convergent validity

The associations between DHI‒CK and the comparators were examined via Spearman’s robust rank correlation^36,41^. Referenced values for the associations were <0.3, >0.3 < 0.5, >0.5 < 0.7, and >0.7 for weak, moderate, adequate, and high correlations, respectively^42,43^.

#### Discriminating validity

The ability of the four scales to discriminate between different groups and subgroups; that is, patient/healthy groups and the patients’ subgroups were examined by employing the following two methods:

1. The ROC curve. Concerning the AUC, we followed Hosmer and colleagues^44^, with referenced values as follows: AUC = 0.5, 0.5 < AUC < 0.7, 0.7 ≤ AUC < 0.8, 0.8 ≤ AUC < 0.9, and AUC > 0.9 suggested no, poor, acceptable, excellent, and outstanding discrimination, respectively. The Youden indices and their associated criterion values for the scales were estimated.
2. With a significance level of 5%, we utilised the Mann-Whitney U test to examine discriminating validity. Since the shape and the distribution of the scales between the patient and the healthy groups were dissimilar, we compared mean ranks instead of medians; however, for patients’ subgroups, we compared the medians because the shapes were similar^41^.

#### Software

SPSS 21 (IBM, Armonk, NY, USA) was used for all steps of the analysis except for rhoC and rhoA, which were determined by SmartPLS 3^45^. Data related to the ROC curve analysis (Table 5) were obtained from MedCalc for Windows, version 19.0.3 (MedCalc Software, Ostend, Belgium).

## Supplementary information

Supplementary Tables S1, S2, and S3

Dataset 1

## Data Availability

The authors confirm that the supplemental tables and data supporting the findings of this study are available within the supplementary materials.
