# Supplementary material for: Reliability and validity of a Central Kurdish version of the Dizziness Handicap Inventory
Source: Sci Rep. 2019 Jun 12;9:8542. doi: 10.1038/s41598-019-45033-1 (PMC6562039; doi:10.1038/s41598-019-45033-1)
Supplement: Supplementary file 1 — Supplementary Tables S1, S2, and S3 [file 41598_2019_45033_MOESM1_ESM.pdf]

Reliability and validity of a Central Kurdish version of the Dizziness Handicap Inventory

Sherko Saeed F. Zmnako and Yousif Ibrahim Chalabi

Supplementary Table S1. Skewness, kurtosis, and internal consistency variables with and without item–E15

	DHI–CK (n = 301)							
	Skewness[a]	Kurtosis[a]	**Alpha if item deleted**				***AIID***	***CI–TC***
			DHI–P	DHI–E	DHI–F	DHI–T	*DHI–T*	*DHI–T*
P1- Looking up	0.04	-1.60	0.661			0.872	*0.870*	*0.32*
E2- Being frustrated	-1.98	3.00		0.736		0.870	*0.868*	*0.42*
F3- Restricting travel	0.25	-1.73			0.674	0.866	*0.865*	*0.51*
P4- Walk via supermarket aisle	1.03	-0.68	0.693			0.869	*0.867*	*0.43*
F5- Getting out or into bed	-0.26	-1.22			0.729	0.872	*0.870*	*0.33*
F6- Restricting social activities	0.14	-1.70			0.678	0.866	*0.864*	*0.53*
F7- Reading difficulties	0.70	-1.25			0.728	0.873	*0.871*	*0.29*
P8- Sports-like activities	0.48	-1.51	0.686			0.866	*0.864*	*0.52*
E9- Afraid to leave home alone	0.57	-1.55		0.724		0.867	*0.865*	*0.50*
E10- Embarrassment	1.96	2.17		0.735		0.868	*0.867*	*0.45*
P11- Quick head movement	-0.39	-1.50	0.635			0.868	*0.866*	*0.47*
F12- Avoid heights	-0.25	-1.78			0.734	0.873	*0.871*	*0.32*
P13- Turning over in bed	0.17	-1.65	0.688			0.872	*0.870*	*0.34*
F14- heavy housework	-0.12	-1.89			0.679	0.865	*0.863*	*0.54*
E15- considered intoxicated	3.31	9.65		0.750		0.872		
F16- Difficult to go for a walk	0.74	-1.26			0.682	0.864	*0.862*	*0.60*
P17- Sidewalk walking	0.44	-1.36	0.710			0.870	*0.868*	*0.40*
E18- Concentration difficulties	-0.05	-1.64		0.758		0.872	*0.870*	*0.34*
F19- Walking in the dark	1.45	0.42			0.719	0.871	*0.869*	*0.35*
E20- Fear of being alone	1.36	-0.26		0.726		0.867	*0.865*	*0.48*
E21- Feelings handicapped	0.16	-1.75		0.704		0.868	*0.866*	*0.45*
E22- Stress on relationships	0.71	-0.96		0.717		0.867	*0.866*	*0.48*
E23- Being depressed	-0.74	-1.15		0.710		0.870	*0.868*	*0.39*
F24- Responsibilities issue	0.38	-1.44			0.669	0.863	*0.861*	*0.63*
P25- Bending over	-0.42	-1.30	0.657			0.868	*0.866*	*0.46*
**Cronbach's alpha**			**0.709**	**0.752**	**0.725**	**0.873**		
Values when item–E15 deleted								
Cronbach's alpha				*0.751*			*0.872*	
AIC				*0.27*			*0.22*	
RhoC				*0.82*				
RhoA				*0.76*				

Notes: For simplicity items shortened; [a]Absolute values of skewness and kurtosis; Alphas are of three decimal places to be compared with Alpha when any item deleted; Alphas of the scales are in bold; Values in italic were generated when item-E15 deleted.
Abbrevitions: DHI–CK/P/E/F/T, Dizziness Handicap Inventory–Central Kurdish/Physical/Emotional/Functional/Total; AIID, Alpha If Item Deleted; CI–TC, Corrected Item–Total Correlation; AIC, Average Inter-item Correlation; rhoC, Composite reliability; rhoA, Consistent reliability of the partial least squares.

Supplementary Table S2. Pearson's correlations between the scales and the comparators

	n = 301			n = 290	n = 286
	DHI–P	DHI–E	DHI–F	VAS–T	CTSIB–T
**DHI–P**				0.44	-0.30
**DHI–E**	0.43			0.56	-0.33
**DHI–F**	0.68	0.70		0.56	-0.38
**DHI–T**	0.81	0.84	0.93	0.61	-0.40

Note: Correlations mentioned in the hypotheses are in bold.
Abbreviations: DHI–P/E/F/T, Dizziness Handicap Inventory–Physical/Emotional/Functional/Total; VAS–T, Visual Analogue Scale–Total; CTSIB–T, Clinical Test of Sensory Interaction and Balance–Total.

Supplementary Table S3. Dizziness handicap inventory-Central Kurdish version

ژمارە	پرسیارەکان	بەڵێ	هەندێکجار	نەخێر
**ئاماری کۆسپەکانی گێژبون<br>Dizziness Handicap Inventory-Central Kurdish version (DHI-CK)**				
تێ بینی: لەم فۆرمەدا مەبەست لە وشەی (کێشە) بریتی یە لەو نەخۆشیە یە یان سکاڵایەی کە بەهۆیەوە سەردانی پزیشکت کردوە (گێژی، وڕی، سەرەسوڕە).				
**ژمارە**	**پرسیارەکان**	**بەڵێ**	**هەندێکجار**	**نەخێر**
P1	ئایا کێشەکەت زیاد دەبێت، ئەگەر سەیری سەرەوە بکەیت؟			
E2	بەهۆی ئەم کێشەیەت، هەست بە بێزاری دەکەیت؟			
F3	بەهۆی ئەم کێشەیەت، سەفەرکردنت سنوردار کردوە، بۆ مەبەستی ئیشوکار یان حەوانەوە؟			
P4	ئایا ڕۆشتن بە ڕاڕەوەکانی سوپەرمارکێت دا کێشەکانت بۆ زیاد دەکات ؟			
F5	بەهۆی ئەم کێشەیەت، گرفتت هەیە بۆ چونە ناو یان هاتنە دەرەوە لە جێگەدا؟			
F6	ئایا ئەم کێشەیەت، تاڕادەیەکی زۆر چالاکیە کۆمەڵایەتی یەکانت سنوردارەدەکات، وەکو ڕۆشتن بۆنانخواردن لە دەرەوە یان بەشداریکردن لە شایی و ئاهەنگ و پرسەکان؟			
F7	بەهۆی ئەم کێشەیەت، گرفتی خوێندنەوەت هەیە ؟			
P8	ئایا کێشەکەت زیاد دەبێت، ئەگەر چالاکی ئەنجام بدەیت وەک: وەرزش یان ئیشوکاری ماڵ وەک گسکدان و لابردنی قاپ یان شتومەک؟			
E9	بەهۆی ئەم کێشەیەت، دەترسیت بە تەنیا لە ماڵ بچیتە دەرێ، بە بێ ئەوەی کەسێکت لەگەڵدا بێت؟			
E10	بەهۆی ئەم کێشەیەت، لە بەردەم کەسانی تر هەستت بە ئیحراج بون کردوە؟			
P11	ئایا کێشەکەت زیاد دەبێت، ئەگەر بە خێرایی سەر بجوڵێنیت؟			
F12	بەهۆی ئەم کێشەیەت، خۆت بەدور دەگریت لە شوێنە بەرزەکان؟			
P13	ئایا کێشەکەت زیاد دەبێت، ئەگەر ئەمدیو و ئەودیو بکەیت لە جێگەدا؟			
F14	بەهۆی ئەم کێشەیەت، ئایا زەحمەتە بۆ تۆ کاری قورسی ناوماڵ یان باخداری بکەیت؟			
E15	بەهۆی ئەم کێشەیەت، دەترسی خەلک وابزانێ تۆ مەستیت یان سەرخۆشیت ؟			
F16	بەهۆی ئەم کێشەیەت، زەحمەتە بەتەنها بڕۆیتە دەرەوە بۆ پیاسە؟			
P17	ئایا کێشەکەت زیاد دەبێت، ئەگەر بەسەر شۆستەدا بڕۆیت؟			
E18	بەهۆی ئەم کێشەیەت، ئایا زەحمەتە تەرکیز بکەیت؟			
F19	بەهۆی ئەم کێشەیەت، ئایا زەحمەتە لە تاریکیدا بە ناو ماڵەکەتدا بگەڕێیت؟			
E20	بەهۆی ئەم کێشەیەت، ئایا دەترسیت بەتەنها لە ماڵ بیت؟			
E21	بەهۆی ئەم کێشەیەت، ئایا هەست دەکەیت پەکتکەوتوە؟			
E22	ئایا ئەم کێشەیەت فشاری خستۆتە سەر پەیوەندیەکانی تۆ لەگەڵ ئەندامانی خێزانەکەت یان هاوڕێکانت؟			
E23	بەهۆی ئەم کێشەیەت. ئایا تۆ دڵتەنگیت؟			
F24	ئایا ئەم کێشەیەت، کاری کردۆتە سەر ئیشوکارت یان بەرپرسیاریەتی تۆ لە ماڵەوە؟			
P25	ئایا کێشەکەت زیاد دەبێت، لە کاتی خۆنوشتاندنەوە؟			
